# Rare Case of Trichomonal Peritonitis

**DOI:** 10.3201/eid1707.100892

**Published:** 2011-07

**Authors:** Crystal A. Zalonis, Allan Pillay, William Secor, Burt Humburg, Robert Aber

**Affiliations:** Author affiliations: Penn State Milton S. Hershey Medical Center, Hershey, Pennsylvania, USA (C.A. Zalonis, B. Humburg, R. Aber);; Centers for Disease Control and Prevention, Atlanta, Georgia, USA (A. Pillay, W. Secor)

**Keywords:** Tritrichomonas foetus, Tritrichomonas suis, immunocompromised, peritonitis, parasite, human, letter

**To the Editor:** Trichomonads are flagellated protozoa with several species capable of infecting or colonizing humans. The most common, *Trichomonas vaginalis*, causes genitourinary infection in adults and, rarely, respiratory infections in premature neonates of infected mothers. *Pentatrichomonas hominis* has been isolated from the human gastrointestinal tract, and *Trichomonas tenax*, a human oropharynx commensal, has caused empyema in immunocompomised persons. Rare cases of human peritonitis caused by trichomonads have been reported.

Some trichomonads, including *Tritrichomonas foetus* and *Tritrichomonas suis*, primarily infect and colonize animals. Although they were previously thought to be different species, current molecular and biologic evidence suggests they are indistinguishable (*1*). *T*. *foetus* (synonym *T. suis*) causes genitourinary infection in cattle and diarrhea in cats and colonizes the gastrointestinal tract of swine.

We report *T. foetus* peritonitis in a 52-year-old man with common variable immunodeficiency, rheumatoid arthritis, splenectomy, and cryptogenic cirrhosis. In June 2007, he was admitted with peritonitis to a community hospital in Pennsylvania, United States. He lived on a farm that had swine, horses, and cats. Exposure to cattle was unknown. He denied having a history of sexually transmitted infections or high-risk sexual behavior.

Initial examination showed paracentesis fluid with numerous motile, flagellated organisms consistent with trichomonads. Bacterial fluid cultures had no growth. Despite receiving antimicrobial drugs (including metronidazole 500 mg intravenously every 6 hours), he became increasingly ill over the following 72 hours with hypotension, acute renal failure, and metabolic acidosis, which required transfer to Penn State Milton S. Hershey Medical Center (Hershey, PA, USA) for further care.

Upon arrival, the man was afebrile but hypotensive and tachycardic. Abdominal examination showed ascites, decreased bowel sounds, and diffuse tenderness. Genitourinary examination results were normal. Repeat paracentesis demonstrated numerous motile trichomonads. Urinalysis and routine cultures of peritoneal fluid and blood were negative. Computed tomography of the abdomen and pelvis showed edematous bowel, ascites, and peritonitis. His condition deteriorated during the following 48 hours. Despite ongoing treatment with broad-spectrum antimicrobial drugs (including metronidazole 500 mg intravenously every 6 hours), he died of multiorgan failure.

Autopsy showed peritonitis with copious intraabdominal exudate and peripancreatic and perigastric abscesses. No intestinal perforation or genitourinary abnormalities were noted. No portal of entry for peritoneal infection was identified. Premortem abdominal fluid samples were sent to the Centers for Disease Control and Prevention (Atlanta, GA, USA) for analysis.

DNA was extracted from the trichomonad culture and peritoneal fluid by using the QIAamp DNA mini-kit (QIAGEN, Valencia, CA, USA). PCR testing for *T. vaginalis* was performed (*2*). PCR for *T. foetus* was performed by using primers TFR3 and TFR4 with thermocycling conditions outlined previously (*3*). PCR was performed in a 50-µL reaction volume with 1 µL of deoxynucleoside triphosphate mix (12.5 mmol/L each of dATP, dCTP, dGTP, and 5 mmol/L of dUTP; Applied Biosystems, Foster City, CA, USA), 5 µL of MgCl_2_ (25 mmol/L; Applied Biosystems), 0.2 µM each primer, 2.5U of AmpliTaq Gold polymerase (Applied Biosystems), 5 µL of 10× PCR buffer (Applied Biosystems), and 5 µL of DNA. PCR products were analyzed on an Agilent 2100 Bioanalyzer (Agilent Technologies, Palo Alto, CA, USA). Amplicons were purified with the QIAquick PCR purification kit (QIAGEN) and directly sequenced with PCR primers on an ABI 3130-XL Genetic Analyzer (Applied Biosystems). Sequences were assembled and aligned with Lasergene software (DNASTAR, Inc., Madison, WI, USA) and deposited in GenBank (accession no. HQ849063).

Metronidazole sensitivity was tested with methods previously described (*4*). The patient’s trichomonads had minimal lethal concentration (MLC) of 3.1 μg/mL for metronidazole, similar to MLCs of the known metronidazole–sensitive *T. vaginalis* isolate. *T. vaginalis* metronidazole MLCs >50 μg are associated with resistance (*5*).

PCR performed by using primers TFR3/4 produced a 348-bp amplicon with DNA extracted from peritoneal fluid and culture (Figure). Comparison of DNA sequence from the parasite to GenBank sequences showed 100% identity with cattle isolates of *T. foetus.*

**Figure:**
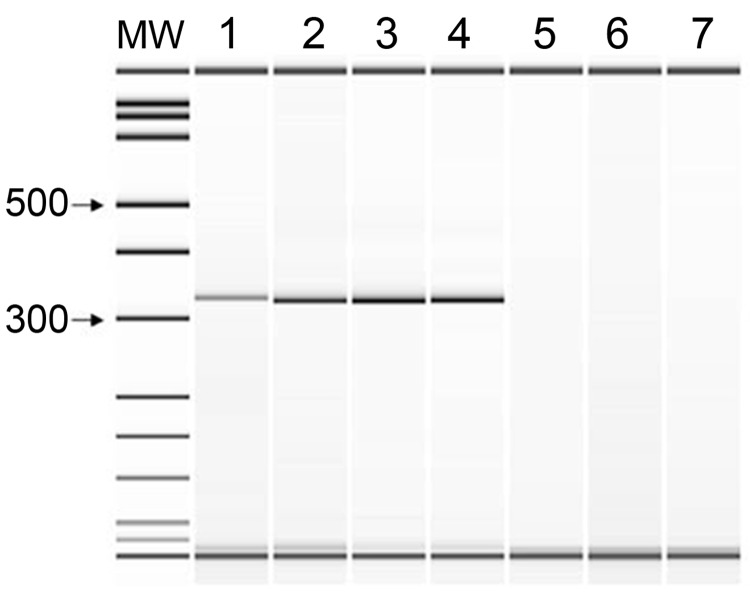
PCR amplification with primers TFR3 and TFR4. Lane 1, *Tritrichomonas foetus* ATCC 30231; lane 2, peritoneal fluid specimen; lane 3, culture of peritoneal fluid; lane 4, *T. suis* ATCC 30167; lane 5, *Pentatrichomonas hominis*; lane 6, *Trichomonas tenax*; lane 7, no template control. MW, molecular size standard. Values at left are bp.

Two human cases of *T. foetus* infection have been reported. *T. foetus* was identified by PCR in the respiratory tract of a patient with AIDS and pneumonia (*6*) and by microscopy in cerebrospinal fluid from a hematopoetic stem-cell transplant recipient with fatal meningoencephalitis (*7*). The latter patient described by Okamoto et al. had a history of trichomonads in a urine sample before transplantation and clinical epididymitis when meningoencephalitis was diagnosed, leading to their conclusion that his infection was genitourinary in origin. The patient we report had no apparent signs of genitourinary infection.

Human trichomonal peritonitis has been reported (*8**–**10*). Straube et al. described a 54-year-old man with common variable immunodeficiency and cirrhosis. Peritoneal fluid contained numerous trichomonads, identified as *T. faecalis* (syn *T. equi*), an intestinal commensal in horses. This patient died shortly after diagnosis. These authors did not describe animal exposures. The patient we report had animal contact. We found no reported cases of *T. foetus* peritonitis.

Two reported patients with trichomonal peritonitis recovered after treatment with metronidazole. Thus, we propose initial treatment with metronidazole in patients with trichomonal peritonitis but confirmation of species and sensitivity to antimicrobial drugs is essential.
